# Primary Brain Tumor Research Productivity in Southeast Asia and Its Association With Socioeconomic Determinants and Burden of Disease

**DOI:** 10.3389/fonc.2020.607777

**Published:** 2020-12-23

**Authors:** Mark Willy L. Mondia, Adrian I. Espiritu, Roland Dominic G. Jamora

**Affiliations:** ^1^Division of Adult Neurology, Department of Neurosciences, College of Medicine and Philippine General Hospital, University of the Philippines Manila, Manila, Philippines; ^2^Department of Clinical Epidemiology, College of Medicine, University of the Philippines Manila, Manila, Philippines; ^3^Institute for Neurosciences, St. Luke's Medical Center, Quezon City & Global City, Philippines

**Keywords:** brain tumor, socioeconomic factors, burden of disease, bibliometric analysis, research productivity, Southeast Asia

## Abstract

**Background:**

There is an unmet need to assess research productivity from southeast Asia (SEA) regarding primary central nervous system (CNS) tumors. The country’s economy, landscape of neurology practice, and disease burden are hypothesized to correlate with scientific output. This study aimed to objectively measure the impact of published studies on primary brain tumors in SEA and to assess for correlation with socioeconomic determinants and burden of disease.

**Methods:**

We systematically searched electronic databases for relevant articles from SEA on primary CNS tumor until July 31, 2020. Bibliometric indices were reported and subjected to correlational analysis with population size, gross domestic product (GDP) per capita, percentage (%) GDP for research and development (R&D), total number of neurologists, disease incidence, deaths, and disability-adjusted life years.

**Results:**

A total of 549 articles were included, consisting primarily of case reports (n=187, 34.06%) and discussed gliomas (n=195, 35.52%). Singapore published the most number of the articles (n=246, 44.8%). Statistical analysis showed a positive correlation between %GDP for R&D and total publication. Additionally, negative relationships were noted between burden of disease and total neurologist with most bibliometric indices. However, GDP per capita was not correlated with measures for research productivity.

**Conclusion:**

The low impact of scientific output on primary CNS tumors in SEA does not address the growing epidemiology and burden of this disease. An increase in the GDP growth and financial and manpower investment to R&D may significantly improve research productivity in SEA.

## Introduction

The incidence of CNS cancer in developing nations has seen a percentage increase change from as low as 15% to as high as 80% in comparison to data last 2009 that cited highest incidence in developed countries (1–3). At present, the world population is estimated at 7.8 billion with Indonesia, the Philippines, Vietnam, and Thailand included in the top 20 largest countries by population (4). These southeast Asian (SEA) countries are projected to grow by 4.9% in terms of gross domestic product (GDP) by 2024 (5).

The global variability of CNS tumors have been attributed to differences in environmental factors, genetic susceptibility, cultural practices, and resources for accurate diagnosis (6). Multidisciplinary approach including neurologists, neurosurgeons, radiation oncologists, and neuropathologists is the usual practice in treating patients with CNS tumors; however most data on regional practice mostly come from Western countries (7). Most Neuro-oncologists in the United States (US) are neurologists who undergo additional training in Neuro-oncology (8). Neuro-oncology is an emerging neurologic subspecialty wherein Neuro-oncologist provide diagnosis and treatment for patients with brain tumors (8).

It has been shown that accelerating progress in cancer-related research through increasing research and development (R&D) expenditure has the potential to improve the health and quality of life of patients with CNS tumors (9, 10). The clinical impact of a research output can be objectively measured using Alternative Metrics or “almetrics” like PlumX from Plum Analytics. These incorporates traditional bibliometrics like citations alongside electronic-based measures such as downloads, abstract views, online comments, and social media likes, shares, and tweets (11). The goal of almetrics is to present a more complete profile of scholarly impact of a research article (11).

A systematic review on the published articles that utilized data from the National Cancer Database from the United States, determined that brain tumors were part of the top 10 topics published about cancer (12). There has only been one bibliometric study published to date about glioblastomas, which tackled the top 100 most cited journals on glioblastoma multiforme (GBM) from 2001 to 2010 and most studies came from high-impact journals with western authors (13).

Currently, there are no published data about scientific research output measures on brain tumors specific to the SEA region. Therefore, we aimed to provide a systematic review of the bibliometric indices of primary CNS tumor research performance of SEA countries and provide a correlational analysis with socioeconomic determinants and disease burden.

## Methods

This systematic review adhered to the Preferred Reporting Items for Systematic reviews and Meta-Analyses (PRISMA) (14).

### Criteria for Inclusion of Studies

We included human and animal subjects with study designs of randomized controlled trials (RCTs), meta-analysis, systematic reviews, retrospective/prospective cohort studies, case-control studies, cross-sectional studies, case series/reports, in-vivo laboratory studies, and literature reviews. The included studies had at least 1 author affiliated to any of the SEA countries (Brunei, Cambodia, Indonesia, Laos, Malaysia, Myanmar, the Philippines, Singapore, Thailand, Timor-Leste and Vietnam). The articles reported on research domains tackling primary brain tumors, which our study grouped according to the WHO Brain Tumor Classification (15).

### Identification and Selection of Studies

We searched PubMed, Scopus, EMBASE, and Clinicaltrials.gov for relevant articles. The search period began last July 15, 2020. We included indexed articles involving primary CNS tumors in SEA countries between 1980 and July 31, 2020. We used the following general search terms: [central nervous system OR brain OR tumor OR neoplasm] AND [Philippines OR Brunei OR Cambodia OR Indonesia OR Lao OR Malaysia OR Myanmar OR Singapore OR Thailand OR Timor-Leste OR Vietnam]. Once duplicates were excluded, we retrieved the full-text of the articles that passed the screening criteria and assessed them for eligibility. We utilized the National Institutes of Health Quality Assessment Tool for case series studies to assess methodological quality when applicable (16). The included studies were then subjected to quantitative and qualitative analysis.

### Relevant Bibliometric Indexes

We used the following bibliometric indices for this review: impact factor (IF), number of overall publications per country, and alternative metrics. We extracted the IF from the Journal Citation Report from Clarivate Analytics (17). This reflects the average citations of an article in the past 2 years. The total number of published articles was tabulated per SEA country. The following alternative metrics were obtained from PlumX metrics (product of Plum Analytics): a) *citations*, which include traditional citation indexes, patent citations, and clinical citations; b) *usage*, composing of clicks, downloads, and views; c) *captures*, which track articles that have been bookmarked, read, exported, and saved in reference programs; d) *mentions*, which quantifies activities in blog posts, comments, reviews, and news media; and e) *social media*, which incorporate Facebook likes, Tweets, and comments on different social media platforms. These indexes reflect bibliometric statistics that correlate research impact of published articles.

### Socioeconomic Factors and Burden of Disease Parameters

We extracted the following data : a) July 2020 population of SEA countries from the World Economic Outlook Database (4); b) GDP per capita and percent allocation of GDP for R&D from the World Bank Database (18); c) total number of neurologist from latest report of the Asian Oceanian Association of Neurology (AOAN) as a surrogate measure for human resources involved in care of CNS tumor patients (19); d) CNS tumor regional burden of disease measures (incidence, death, and disability-adjusted life years; [DALYs]) from the Global Burden of Disease Study of 2016 (1).

### Data Synthesis and Analysis

We extracted the following information from each included study: title, author/s affiliated with institutions based in SEA countries, year of publication, journal name in which the article was published, study design, CNS tumor classification, latest impact factor, and specific topic studied (e.g. pathophysiology, clinical experience, epidemiology, diagnosis, treatment, prognosis).

We analyzed the data using the IBM® SPSS® Statistics for Macintosh Version 24 (Armonk, NYL IBM Corp.). Descriptive statistics was employed wherein qualitative data were evaluated using frequencies and proportions, while continuous data were presented as means and standard deviations. Correlation was determined using Pearson coefficient (*R)*, with statistical significance if *p*-value is < 0.1.

## Results

### Systematic Search of Studies

**Figure 1:**
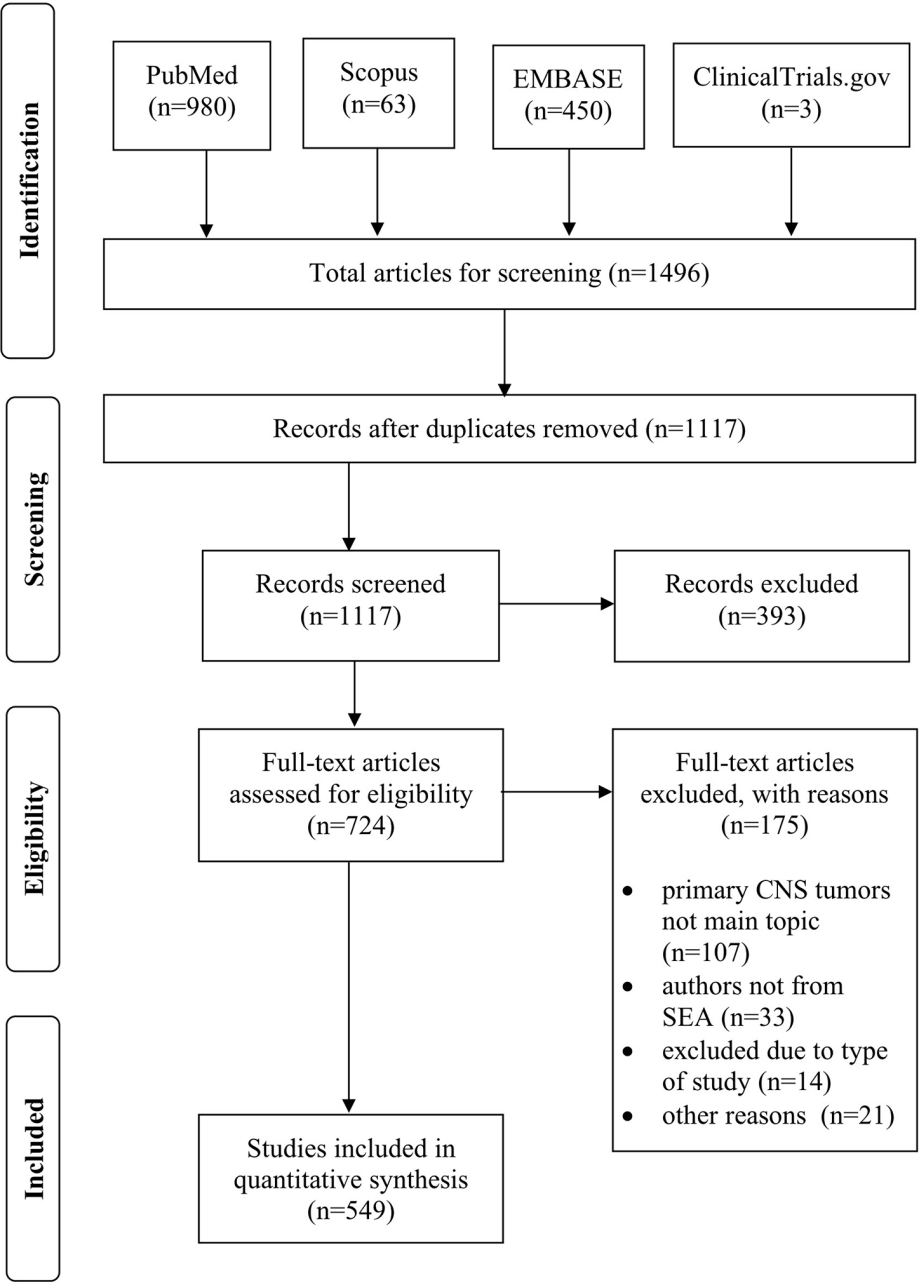
PRISMA flow diagram of the article selection process.

The search strategy yielded a total of 1,496 articles (PubMed: 980; Scopus: 63; EMBASE: 450; ClinicalTrials.gov: 3) (**Figure 1**). After duplicates were moved, 1,117 articles were screened. We excluded 393 articles due to: a) primary CNS tumors were not the main topic; b) no author was affiliated with any institution from SEA countries; c) type of study design; and d) incomplete author affiliation data. Thus, 724 studies were screened for inclusion, with only 549 articles meeting the criteria and included in the analysis.

### Characteristics of Included Studies

In terms of study design, majority were case reports (n=187, 34%), cohort studies (n=159, 29%) and animal/laboratory studies (n=134, 24.4%), followed by literature reviews (n=27, 4.9%), case series (n=26, 4.7%), and cross-sectional design (n=3, 0.6%). There were a limited number of systematic reviews/meta-analyses (n=7, 1.3%), RCT (n=1, 0.2%), and case-controls (n=5, 0.9%). The research focus were on clinical experience (n=204, 37.2%), pathophysiology (n=134, 24.4%), treatment (n=102, 18.6%), diagnosis (n=58, 10.6%), prognosis (n=27, 4.9%), and epidemiology (n=24, 4.4%).

**Figure 2:**
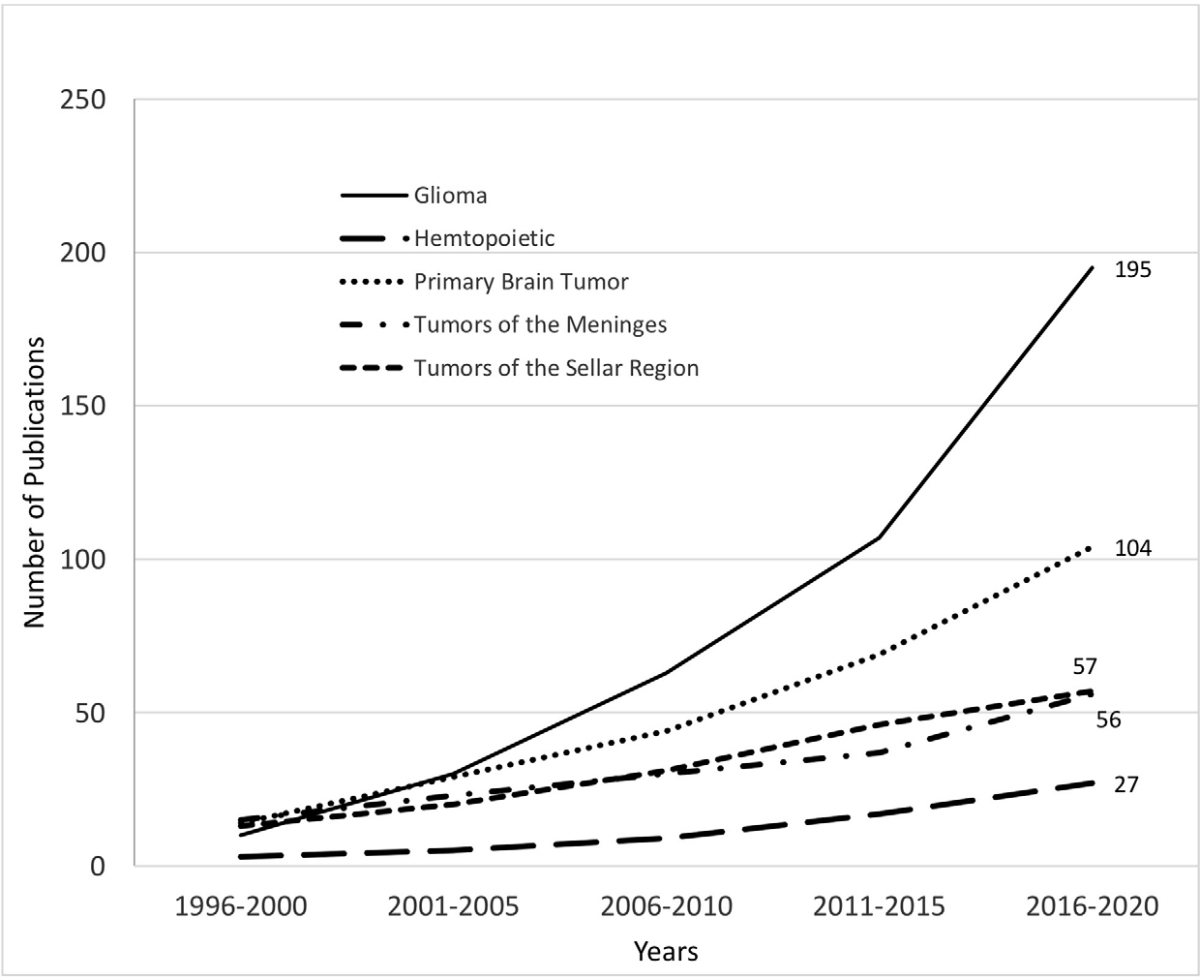
Published journal articles on top topics of primary CNS tumor from the SEA region during 1996 to 2020.

Most of the studies discussed gliomas (n=195, 35.5%) and mixture of primary brain tumors (n=104, 18.9%). Tumors of the sellar region (n=57, 10.4%) and of the meninges (n=56, 10.2%) were also commonly reported. Hematopoietic tumors particularly primary CNS lymphomas (n=27, 4.9%), germ cell tumors (n=23, 4.2%), embryonal tumors (n=21, 3.8%), cysts and tumor-like lesions (n=18, 3.3%), and non-meningothelial tumors of the meninges (n=15, 2.73%) had comparable frequencies. The least reported tumor types discussed were: tumors of the cranial nerves and spinal nerves (n=8, 1.46%), neuronal and mixed neuronal-glial tumors (n=6, 1.09%), local extensions from regional tumors (n=4, 0.73%), ependymal cell tumors (n=2, 0.36%), neuroepithelial tumors (n=1, 0.18%), and tumors of the choroid plexus (n=1, 0.18%). There were also reported non-neuronal/glial cell tumors arising primarily in the CNS without evidence of metastasis (n=11, 2%). There was a steadily increasing number of research output starting 1996 with noted exception from Brunei, Cambodia, Lao, Myanmar, and Timor-Leste (**Figure 2**).

The age distribution of subjects involved in the included studies had the following demographics: adult population (n=337, 61.4%), pediatric age group (n=71, 12.9%), both adult and pediatric patients (n=6, 1.1%). Some articles did not specify age of population of interest (n=135, 24.6%).

### Primary Brain Tumor Research Output: Top Journals and Southeast Asian Institutions

The top journals that published articles on primary CNS tumors from the SEA region were: *Journal of the Medical Association of Thailand* (n=61, 11.1%), *The Medical Journal of Malaysia* (n=27, 4.9%), *Singapore Medical Journal* (n=26, 4.7%), *Journal of Clinical Neurosciences* (n=21, 3.8%), *Annals of the Academy of Medicine Singapore* (n=19, 3.5%), and *World Neurosurgery* (n=15, 2.7%) (**Table 1**). One retrospective cohort study on medulloblastomas from Singapore was published in *The Lancet Oncology*, which had the highest IF (33.752) among all included articles (20). Five articles were published in discontinued journals. The authors affiliated with the following SEA institutions had the highest number of publications on primary brain neoplasms: *National University of Singapore* – Singapore (n=97 articles, 17.7%), *Mahidol University* – Thailand (n=58, 10.6%), *National Neuroscience Institute, Tan Tock Seng Hospital* – Singapore (n=57, 10.4%), *Chulalongkorn University* – Thailand (n=51, 9.3%), and *Singapore General Hospital* – Singapore (n=48, 8.7%). There were 77 institutions (public: 57, private: 20) from SEA that published on primary brain tumors. There are 25 institutions with existing Neurology residency programs and only 2 institutions offering a fellowship in neurooncology: one public institute in Singapore and one private hospital in the Philippines (**Table 2**).

**Table 1:** Journals where studies on primary central nervous system tumors from Southeast Asia were published (n=549).

Journal Name	Frequency, n (%)	Impact factor
*Journal of the Medical Association of Thailand*	61 (11.11%)	0.05
*The Medical Journal of Malaysia*	27 (4.92%)	0.26
*Singapore Medical Journal*	26 (4.74%)	1.359
*Journal of Clinical Neuroscience*	21 (3.83%)	1.76
*Annals of the Academy of Medicine Singapore*	19 (3.46%)	1.533
*World Neurosurgery*	15 (2.73%)	1.829
*Journal of Neuro-Oncology*	9 (1.64%)	1.86
*Journal of Neurosurgery*	8 (1.46%)	3.968
*Biomaterials*	7 (1.28%)	10.317
*British Journal of Neurosurgery*	6 (1.09%)	1.29
*Cancer Research*	6 (1.09%)	9.727
*Child's Nervous System*	6 (1.09%)	1.32
*Neuropathology*	6 (1.09%)	1.758
*Acta Neurochirurgica*	5 (0.91%)	1.817
*American Journal of Neuroradiology*	5 (0.91%)	3.381
*BMJ Case Reports*	5 (0.91%)	0.44
*Clinical Radiology*	5 (0.91%)	2.118
*Journal of Controlled Release*	5 (0.91%)	7.727
*Malaysian Journal of Medical Sciences*	5 (0.91%)	0.39
*Pharmaceutical Research*	5 (0.91%)	3.242
*Asian Journal of Surgery*	4 (0.73%)	1.838
*Asian Pacific Journal of Cancer Prevention*	4 (0.73%)	1.23
*Clinical Neurology and Neurosurgery*	4 (0.73%)	1.53
*Clinical Neuropathology*	4 (0.73%)	1.103
*International Journal of Radiation Oncology Biology Physics*	4 (0.73%)	5.859
*Magnetic Resonance Imaging*	4 (0.73%)	3.94
*Neuro-oncology*	4 (0.73%)	10.427
*Neurosurgery*	4 (0.73%)	4.853
*Pathology*	4 (0.73%)	3.744
*PLoS ONE*	4 (0.73%)	2.74
*Acta Cytologica*	3 (0.55%)	1.226
*Asia-Pacific Journal of Clinical Oncology*	3 (0.55%)	2.012
*BMC Cancer*	3 (0.55%)	3.15
*Journal of Surgical Oncology*	3 (0.55%)	2.771
*Journal of the Medical Association of Thailand*	3 (0.55%)	0.05
*Neurological Research*	3 (0.55%)	2.401
*Neurology*	3 (0.55%)	8.77
*Pediatric Blood and Cancer*	3 (0.55%)	2.355
*Pituitary*	3 (0.55%)	3.954
*Proceedings of the National Academy of Sciences of the United States of America*	3 (0.55%)	9.412
*Stereotactic and Functional Neurosurgery*	3 (0.55%)	1.635
*Advanced Materials*	2 (0.36%)	27.398
*Biomedicine and Pharmacotherapy*	2 (0.36%)	4.545
*Brain Pathology*	2 (0.36%)	5.568
*Brain Tumor Pathology*	2 (0.36%)	2.348
*Cancer Letters*	2 (0.36%)	7.36
*Cellular and Molecular Neurobiology*	2 (0.36%)	3.606
*Clinical Cancer Research*	2 (0.36%)	10.107
*Computational and Mathematical Methods in Medicine*	2 (0.36%)	1.77
*Endocrine*	2 (0.36%)	3.235
*Experimental Biology and Medicine*	2 (0.36%)	3.139
*In Vivo*	2 (0.36%)	1.541
*International Journal of Oncology*	2 (0.36%)	3.899
*Journal of Neurology, Neurosurgery and Psychiatry*	2 (0.36%)	8.689
*Journal of Neuropathology and Experimental Neurology*	2 (0.36%)	2.923
*Journal of Neurosciences in Rural Practice*	2 (0.36%)	0.74
*Journal of Radiology Case Reports*	2 (0.36%)	0.28
*Malaysian Journal of Pathology*	2 (0.36%)	0.477
*Medical Journal of Indonesia*	2 (0.36%)	0.17
*Medical Molecular Morphology*	2 (0.36%)	2.429
*Molecular Cancer*	2 (0.36%)	15.302
*Nature Communications*	2 (0.36%)	12.121
*Neuroradiology*	2 (0.36%)	2.467
*Oncogene*	2 (0.36%)	7.971
*Oncology Reports*	2 (0.36%)	3.417
*Oncotarget*	2 (0.36%)	3.71
*Otolaryngologic Clinics of North America*	2 (0.36%)	1.791
*Pediatric Neurology*	2 (0.36%)	2.89
*Pediatrics International*	2 (0.36%)	1.139
*Phytomedicine*	2 (0.36%)	4.268
*Surgical Neurology*	2 (0.36%)	0.59
*Surgical Oncology*	2 (0.36%)	2.521
*Academic Radiology*	1 (0.18%)	2.488
*ACS Nano*	1 (0.18%)	14.588
*Acta Medica Indonesiana*	1 (0.18%)	0.38
*Acta Neurologica Belgica*	1 (0.18%)	1.989
*Acta Neuropathologica*	1 (0.18%)	14.251
*Acta Oncologica*	1 (0.18%)	3.701
*Advances in Experimental Medicine and Biology*	1 (0.18%)	2.45
*American Journal of Clinical Pathology*	1 (0.18%)	2.094
*American Journal of Nuclear Medicine and Molecular Imaging*	1 (0.18%)	1.087
*American Journal of the Medical Sciences*	1 (0.18%)	1.911
*American Society of Clinical Oncology Educational Book*	1 (0.18%)	2.46
*Analytical and Bioanalytical Chemistry*	1 (0.18%)	3.637
*Analytical Chemistry*	1 (0.18%)	6.785
*Andrologia*	1 (0.18%)	1.951
*Annales Paediatrici*	1 (0.18%)	Discontinued
*Annals of Clinical and Translational Neurology*	1 (0.18%)	4.87
*Annals of Diagnostic Pathology*	1 (0.18%)	1.877
*Annals of Hematology*	1 (0.18%)	2.904
*Annals of the New York Academy of Sciences*	1 (0.18%)	4.728
*Anti-Cancer Agents in Medicinal Chemistry*	1 (0.18%)	2.049
*Anticancer Research*	1 (0.18%)	1.994
*Antioxidants and Redox Signaling*	1 (0.18%)	6.323
*Asian Journal of Neurosurgery*	1 (0.18%)	0.905
*Asian Oceanian Journal of Radiology*	1 (0.18%)	Discontinued
*Australasian Radiology*	1 (0.18%)	0.86
*BioEssays*	1 (0.18%)	4.627
*BioMed Research International*	1 (0.18%)	2.276
*Biotechnology and Bioengineering*	1 (0.18%)	4.002
*BMC Complementary and Alternative Medicine*	1 (0.18%)	2.833
*BMC Medical Imaging*	1 (0.18%)	1.622
*BMC Pediatrics*	1 (0.18%)	1.983
*Brain & Development*	1 (0.18%)	1.504
*British Journal of Radiology*	1 (0.18%)	2.196
*Canadian Family Physician*	1 (0.18%)	3.112
*Canadian Medical Association Journal*	1 (0.18%)	7.744
*Cancer*	1 (0.18%)	6.37
*Cancer & Chemotherapy*	1 (0.18%)	3.2
*Cancer Cell*	1 (0.18%)	26.602
*Cancer Detection and Prevention*	1 (0.18%)	3.68
*Cancer Gene Therapy*	1 (0.18%)	4.534
*Cancer Medicine*	1 (0.18%)	3.491
*Cell Reports*	1 (0.18%)	7.7
*Cephalalgia*	1 (0.18%)	4.868
*Child Neuropsychology*	1 (0.18%)	2.405
*Chinese Clinical Oncology*	1 (0.18%)	1.33
*Clinical Imaging*	1 (0.18%)	1.109
*Clinical Neuroradiology*	1 (0.18%)	3.183
*CNS Oncology*	1 (0.18%)	1.69
*Colloids and Surfaces B: Biointerfaces*	1 (0.18%)	4.389
*Computer Methods and Programs in Biomedicine*	1 (0.18%)	3.632
*Diagnostic Pathology*	1 (0.18%)	2.335
*Drug Delivery and Translational Research*	1 (0.18%)	2.664
*EMBO Molecular Medicine*	1 (0.18%)	8.821
*Endocrine Practice*	1 (0.18%)	3.869
*Endocrinology*	1 (0.18%)	3.934
*European Journal of Neurology*	1 (0.18%)	4.516
*European Journal of Pharmaceutical Sciences*	1 (0.18%)	3.616
*European Journal of Radiology*	1 (0.18%)	2.687
*European Neurology*	1 (0.18%)	1.182
*European Radiology*	1 (0.18%)	4.101
*Forensic Science International*	1 (0.18%)	2.108
*Frontiers in Bioscience - Scholar*	1 (0.18%)	2.747
*Gan To KagakuRyoho. Cancer & Chemotherapy*	1 (0.18%)	0.05
*Gene*	1 (0.18%)	2.984
*Gene Therapy*	1 (0.18%)	4.128
*Genes Chromosomes and Cancer*	1 (0.18%)	3.444
*Genetics and molecular research*	1 (0.18%)	0.75
*Handbook of Clinical Neurology*	1 (0.18%)	0.9
*Headache*	1 (0.18%)	4.041
*Hematology*	1 (0.18%)	1.65
*Human Gene Therapy*	1 (0.18%)	4.273
*Interdisciplinary Neurosurgery: Advanced Techniques and Case Management*	1 (0.18%)	0.34
*International Journal of Clinical and Experimental Pathology*	1 (0.18%)	0.159
*International Journal of Gynaecology and Obstetrics*	1 (0.18%)	2.216
*International Journal of Molecular Sciences*	1 (0.18%)	4.556
*International Journal of Pharmaceutics*	1 (0.18%)	4.845
*International Journal of STD and AIDS*	1 (0.18%)	1.07
*Journal Francais d'Ophthalmologie*	1 (0.18%)	0.636
*Journal of Biomaterials Science, Polymer Edition*	1 (0.18%)	2.69
*Journal of Biomedical Optics*	1 (0.18%)	2.785
*Journal of Biomolecular Screening*	1 (0.18%)	2.37
*Journal of Biophotonics*	1 (0.18%)	3.032
*Journal of Cancer Research and Therapeutics*	1 (0.18%)	1.326
*Journal of Cancer Research and Clinical Oncology*	1 (0.18%)	3.656
*Journal of Cellular Biochemistry*	1 (0.18%)	4.237
*Journal of Clinical Oncology*	1 (0.18%)	32.956
*Journal of Clinical Pathology*	1 (0.18%)	2.46
*Journal of Computer Assisted Tomography*	1 (0.18%)	1.285
*Journal of Ethnopharmacology*	1 (0.18%)	3.69
*Journal of Experimental Medicine*	1 (0.18%)	10.35
*Journal of Forensic Sciences*	1 (0.18%)	1.441
*Journal of Health and Translational Medicine*	1 (0.18%)	0.11
*Journal of Human Genetics*	1 (0.18%)	2.831
*Journal of Medical Case Reports*	1 (0.18%)	0.255
*Journal of Molecular Neuroscience*	1 (0.18%)	2.678
*Journal of Neurological Surgery Part A: Central European Neurosurgery*	1 (0.18%)	0.905
*Journal of Neurology and Neurorehabilitation Research *	1 (0.18%)	0.64
*Journal of Neuroscience Methods*	1 (0.18%)	2.214
*Journal of Neuroscience Nursing*	1 (0.36%)	1.109
*Journal of Neurosurgery: Pediatrics*	1 (0.18%)	2.117
*Journal of Neurosurgical Sciences*	1 (0.18%)	1.645
*Journal of Nutritional Biochemistry*	1 (0.18%)	4.873
*Journal of Oncology Practice*	1 (0.18%)	3.551
*Journal of Orthopaedic Surgery*	1 (0.18%)	1.095
*Journal of Pediatric Endocrinology and Metabolism*	1 (0.18%)	1.278
*Journal of Pediatric Hematology/ Oncology*	1 (0.18%)	1.016
*Journal of Pediatric Neurosciences*	1 (0.18%)	0.27
*Journal of Physiology and Biochemistry*	1 (0.18%)	2.523
*Journal of Radiation Research*	1 (0.18%)	1.95
*Journal of the National Cancer Institute*	1 (0.18%)	11.577
*Medical and Biological Engineering and Computing*	1 (0.18%)	2.36
*Minimally Invasive Neurosurgery*	1 (0.18%)	1.55
*Molecular Biology of the Cell*	1 (0.18%)	3.791
*Molecular Neurobiology*	1 (0.18%)	4.5
*Molecular Oncology*	1 (0.18%)	6.574
*Molecules*	1 (0.18%)	3.267
*Multiple Sclerosis and Related Disorders*	1 (0.18%)	2.889
*Mutation Research - Genetic Toxicology and Environmental Mutagenesis*	1 (0.36%)	2.506
*Nanomedicine*	1 (0.18%)	4.3
*Neurochemical Research*	1 (0.18%)	3.038
*NeuroImage: Clinical*	1 (0.18%)	4.35
*Neurologia Medico-Chirurgica*	1 (0.18%)	1.836
*Neurology India*	1 (0.18%)	2.128
*Neuroradiology Journal*	1 (0.18%)	2.238
*Neuroscience Letters*	1 (0.18%)	2.274
*Neurosurgical Focus*	1 (0.18%)	2.59
*Neurosurgical Review*	1 (0.18%)	2.654
*Oncology Letters*	1 (0.18%)	1.871
*Onkologie*	1 (0.18%)	Discontinued
*Otolaryngology*—*Head and Neck Surgery*	1 (0.18%)	3.848
*Panminerva Medica*	1 (0.18%)	3.467
*Pathobiology*	1 (0.18%)	1.831
*Pediatric Surgery International*	1 (0.18%)	1.688
*Pharmacological Reviews*	1 (0.18%)	17.395
*Philippine Journal of Pediatrics*	1 (0.18%)	Discontinued
*Proceedings of Singapore Healthcare*	1 (0.18%)	0.4
*QJM*	1 (0.18%)	2.529
*Radiology*	1 (0.18%)	7.931
*Seminars in Oncology*	1 (0.18%)	4.213
*Singapore Medical Association*	1 (0.18%)	1.26
*Skeletal Radiology*	1 (0.18%)	1.618
*Southeast Asian Journal of Tropical Medicine and Public Health*	1 (0.18%)	0.18
*Stem Cell Reviews and Reports*	1 (0.18%)	5.316
*Stem Cells*	1 (0.18%)	6.022
*Stem Cells and Development*	1 (0.18%)	3.082
*Survey of Ophthalmology*	1 (0.18%)	4.195
*Technology in Cancer Research and Treatment*	1 (0.18%)	2.074
*The Ceylon Medical Journal*	1 (0.18%)	0.36
*The Journal of Clinical Endocrinology and Metabolism*	1 (0.18%)	5.399
*The Journal of Clinical Investigation*	1 (0.18%)	11.864
*The Journal of Laryngology and Otology*	1 (0.18%)	1.098
*The Journal of Obstetrics and Gynaecology Research*	1 (0.18%)	1.392
*The Journal of the Singapore Paediatric Society*	1 (0.18%)	Discontinued
*The Lancet Oncology*	1 (0.18%)	33.752
*Thyroid*	1 (0.18%)	5.227
*Toxicology in Vitro*	1 (0.18%)	2.959
*Tumor Biology*	1 (0.18%)	3.81
*Tumori*	1 (0.18%)	1.707
*Ultrasound in Obstetrics and Gynecology*	1 (0.18%)	5.571
*Virchows Archive: An International Journal of Pathology*	1 (0.18%)	2.906

**Table 2:** Institutions from southeast Asia that published on primary central nervous system tumors.

Country	Institution	Total Publications	Public or Private^†^	Neurology Residency*
Singapore	*National University of Singapore*	97	Public	Yes
	*National Neuroscience Institute-Tan Tock Seng Hospital*	57	Public	Yes
	*Singapore General Hospital*	48	Public	Yes
	*KK Women's and Children's Hospital*	15	Public	No
	*Agency for Science, Technology and Research*	10	Public	No
	*Institute of Bioengineering and Nanotechnology*	3	Private	No
	*Nanyang Technological University*	3	Public	No
	*Molecular Engineering of Biological and Chemical Systems (MEBCS), Singapore-MIT Alliance*	2	Private	No
	*Alexandra Hospital*	1	Public	No
	*Centre for Forensic Medicine, Health Sciences Authority*	1	Public	No
	*Khoo Teck Puat Hospital*	1	Public	No
	*Laboratory of Cancer Gene Therapy, Cellular and Molecular Research Division*	1	Private	No
	Singapore Institute for Clinical Sciences, Biomedical Sciences Institutes (A*STAR)	2	Private	No
	*Raffles Hospital*	1	Private	No
	*Singapore Bioimaging Consortium*	1	Private	No
	*Singapore National Eye Centre*	1	Public	No
	*Vishuo Biomedical*	1	Public	No
Malaysia	*Universiti Sains Malaysia*	29	Public	Yes
	*University of Malaya*	21	Public	No
	*Universiti Kebangsaan Malaysia, The National University of Malaysia*	21	Public	No
	*University of Nottingham Malaysia Campus*	5	Private	No
	*Hospital Kuala Lumpur*	4	Public	Yes
	*Universiti Sains Malaysia*	8	Public	Yes
	*International Medical University*	3	Private	No
	*Hospital Queen Elizabeth II*	2	Public	No
	*Serdang Hospital*	2	Public	No
	*Universiti Putra Malaysia*	2	Public	Yes
	*University Technology Malaysia*	2	Public	No
	*Hospital Ipoh*	1	Public	No
	*Hospital Putrajaya*	1	Private	No
	*Hospital Umum Sarawak*	1	Public	No
	*Institute for Medical Research*	1	Public	No
	*International Islamic University Malaysia*	1	Public	No
	*Malaysia Medical Centre*	1	Private	No
	*MARA University of Technology Clinical Training Centre*	1	Public	No
	*Normah Medical Specialist Center*	1	Private	No
	*Pantai Cheras Medical Center*	1	Public	No
	*Penang General Hospital*	1	Public	Yes
	*SEGi University*	1	Private	No
	*Universiti Malaysia Sarawak*	2	Public	No
	*Universiti Teknologi MARA*	1	Public	No
Thailand	*Mahidol University*	58	Public	Yes
	*Chulalongkorn University*	51	Public	No
	*Prince of Songkla University*	10	Public	No
	*Chiang Mai University*	9	Public	Yes
	*Khon Kaen University*	6	Public	Yes
	*Prasat Neurological Institute*	5	Public	Yes
	*Thammasat University*	3	Public	No
	*Faculty of Pharmaceutical Sciences and Melatonin Research Group*	1	Private	No
	*Maha Vajiralongkorn Thanyaburi Hospital*	1	Public	No
	*Ministry of Public Health*	1	Public	No
	*Naresuan University*	1	Public	No
	*National Nanotechnology Center (NANOTEC), National Science and Technology Development Agency (NSTDA)*	1	Public	No
	*Navamindradhiraj University*	1	Public	Yes
	*Rangsit University*	1	Private	No
	*Ratchaburi Hospital*	1	Public	No
	*Royal Thai Army Institute of Pathology*	1	Public	No
	*Srinakharinwirot University*	1	Public	No
	*Suranaree University of Technology*	1	Public	No
Indonesia	*University of Indonesia*	5	Private	No
	*Diponegoro University*	3	Public	No
	*Airlangga University*	2	Public	Yes
	*Universitas Padjadjaran*	2	Public	Yes
	*Dr. Sardjito General Hospital*	1	Public	Yes
	*Gadjah Mada University*	1	Private	No
	*Padjadjaran University*	1	Public	Yes
	*Pelita Harapan University*	1	Private	No
Philippines	*Philippine General Hospital, University of the Philippines Manila*	6	Public	Yes
	*East Avenue Medical Center*	1	Public	Yes
	*Fatima College of Medicine, Fatima Medical Science Foundation, Inc*.	1	Private	No
	*St. Luke's Medical Center ^‡^*	1	Private	Yes
	*University of the East Ramon Magsaysay Memorial Medical Center*	1	Private	Yes
	*University of the Philippines Diliman*	1	Public	No
Vietnam	*Ho Chi Minh University of Medicine and Pharmacy*	5	Public	Yes
	*Bach Mai Hospital*	2	Public	Yes
	*Cho Ray Hospital*	1	Public	Yes
	*University Hospital Viet Tiep*	1	Public	Yes

^†^Institutions were individually searched via their official websites to determine their type of funding.

*Adapted from Sy et al. (21) where applicable.

^‡^Institution with an existing fellowship in Neurooncology.

### Research Publication Bibliometric Indices

A total of 549 articles about primary CNS tumors were published from the SEA region: 246 (44.8%) from Singapore, 154 (28%) from Thailand, 113 (20.6%) from Malaysia, 15 (2.7%) from Indonesia, 12 (2.2%) from the Philippines, and 9 (1.6%) from Vietnam. Singapore, Thailand, and Malaysia published the most articles as well as had the highest values as reported in PlumX metrics (citations, usage, captures, mentions, and social media) and Scopus citations. No articles were identified from Brunei, Cambodia, Myanmar, and Timor-Leste (**Table 3**).

**Table 3:** Bibliometric indices for articles on primary CNS tumors published from SEA.

Countries*	Publications (%)	PlumX Metrics					Scopus citations (%)
		Citations (%)	Usage (%)	Captures (%)	Mentions (%)	Social Media (%)	
Singapore	246 (44.81)	3,081 (75.83)	15,523 (62.49)	11,537 (62.96)	44 (78.57)	416 (54.74)	4,708 (71.42)
Thailand	154 (28.05)	606 (14.92)	3,181 (12.8)	3,430 (18.72)	1 (1.79)	137 (18.03)	1,168.48 (17.73)
Malaysia	113 (20.58)	279 (6.87)	4,720 (19)	24,96 (13.62)	5 (8.93)	175 (23.03)	547.39 (8.3)
Indonesia	15 (2.73)	35 (0.86)	150 (0.6)	209 (1.14)	0 (0)	4 (0.53)	70 (1.06)
Philippines	12 (2.19)	36 (0.89)	822 (3.31)	150 (0.82)	0 (0)	4 (0.53)	51 (0.77)
Vietnam	9 (1.64)	26 (0.64)	446 (1.8)	502 (2.74)	6 (10.71)	24 (3.16)	47 (0.71)
TOTAL	549 (100)	4,063 (100)	24,842 (100)	18,324 (100)	56 (100)	760 (100)	6,591.87 (100)

*There were no identified publications on primary tumors of the central nervous system from Brunei, Myanmar, Lao PDR, Cambodia, and Timor-Leste.

SEA, Southeast Asia.

### Southeast Asian Region Socioeconomic Determinants

The latest data from 2019 puts the total population size (in millions) of SEA at 661.91, roughly 9% of the world’s total population (4). Indonesia (n=270.6), Philippines (n=108.1), and Vietnam (n=96.5) had the largest population size, while Singapore (n=5.7), Timor-Leste (n=1.29), and Brunei (n=0.43) had the smallest population size.

In contrast, Singapore (USD 65,233.30), Brunei (USD 31,086.80), and Malaysia (USD 11,414.80) had the highest GDP per capita in SEA, while Timor-Leste (USD 1,294.20) had the lowest GDP per capita. Singapore (1.94%) and Malaysia (1.44%) contributed the most percentage of their GDP to R&D despite having only 100 and 120 neurologists, respectively. Thailand ranked third in terms of %GDP for R&D (1.0%) and number of neurologists (n= 645). Lastly, Cambodia, Lao, and Timor-Leste had the lowest GDP per capita, %GDP for R&D and number of neurologists (**Table 4**).

**Table 4:** SEA socioeconomic factors and bibliometric indices correlational analysis.

Socioeconomic factors		Bibliometric indices	Correlation coefficient (*R*)	*P*-value
***Population***^a^ ***(Million)***		Total Publications	−0.691	0.941
Indonesia	270.62			
Philippines	108.12	Plum X Citations	−0.554	0.308
Vietnam	96.46			
Thailand	69.63	Plum X Usage	−0.651	0.155
Myanmar	54.05			
Malaysia	31.95	Plum X Captures	−0.624	0.163
Cambodia	16.49			
Lao PDR	7.17	Plum X Mentions	−0.546	***0.093^†^***
Singapore	5.70			
Timor-Leste	1.29	Plum X Social Media	−0.706	0.770
Brunei	0.43			
Total	661.91	Scopus Citations	−0.571	0.248
***GDP/ Capita^a^ (USD)***		Total Publications	0.842	0.175
Singapore	65,233.28			
Brunei	31,086.75	Plum X Citations	0.990	0.172
Malaysia	11,414.84			
Thailand	7,808.19	Plum X Usage	0.983	0.187
Indonesia	4,135.57			
Philippines	3,485.08	Plum X Captures	0.976	0.183
Vietnam	2,715.28			
Lao PDR	2,534.90	Plum X Mentions	0.985	0.174
Cambodia	1,643.12			
Myanmar	1,407.81	Plum X Social Media	0.940	0.175
Timor-Leste	1,294.19			
Total	(4,796.78^b^)	Scopus Citations	0.984	0.173
***% GDP for R&D***^a^		Total Publications	0.928	***0.070^†^***
Singapore	1.94			
Malaysia	1.44	Plum X Citations	0.807	0.225
Thailand	1.00			
Vietnam	0.53	Plum X Usage	0.895	0.144
Brunei	0.28			
Indonesia	0.23	Plum X Captures	0.877	0.148
Philippines	0.16			
Cambodia	0.12	Plum X Mentions	0.777	0.268
Lao PDR	0.04			
Myanmar	0.03	Plum X Social Media	0.956	0.110
Timor-Leste	NR			
Total	(0.25^c^)	Scopus Citations	0.825	0.200
***Total Neurologists***^b^		Total Publications	−0.690	0.064^†^
Indonesia	1150			
Vietnam	800	Plum X Citations	−0.592	0.845
Thailand	645			
Philippines	506	Plum X Usage	−0.728	0.213
Malaysia	120			
Singapore	100	Plum X Captures	−0.651	0.244
Myanmar	23			
Cambodia	5	Plum X Mentions	−0.591	***0.024^‡^***
Lao PDR	3			
Brunei	2	Plum X Social Media	−0.756	0.108
Timor-Leste	0			
Total	3354	Scopus Citations	−0.600	0.551
***NP per neurologist***^d^		Total Publications	−0.118	***0.025^‡^***
Singapore	57,035.7			
Thailand	107,946.6	Plum X Citations	−0.326	***0.025^‡^***
Vietnam	120,577.6			
Philippines	213,669.2	Plum X Usage	−0.158	***0.029^‡^***
Brunei	216,642.5			
Indonesia	235,326.6	Plum X Captures	−0.257	***0.028^‡^***
Malaysia	266,248.1			
Myanmar	2,349,800.9	Plum X Mentions	−0.293	***0.025^‡^***
Lao PDR	2,389,818.3			
Cambodia	3,297,308.4	Plum X Social Media	−0.104	***0.025^‡^***
Timor-Leste	NA			
Total	(197,349.7^e^)	Scopus Citations	−0.317	***0.025^‡^***
				

GDP, gross domestic product; R&D, research and development; NP, number of population; NR, no record; NA, not applicable; SEA, Southeast Asia.

a Data obtained from www.data.worldbank.org.

b Total SEA GDP/capita computed by dividing total SEA GDP by total SEA population.

c Median %GDP for R&D.

d Data obtained from Roxas et al. (19).

e Computed by dividing total SEA population by the total neurologists.

^†^Correlation is significant at the 0.1 level (2-tailed).

^‡^Correlation is significant at the 0.05 level (2-tailed).

### Burden of Disease of Primary Central Nervous System Neoplasm in Southeast Asian Nations

In 2016, the total incidence of primary brain tumors in SEA was 15,193 in absolute counts. Incidence and deaths showed similar trends (**Table 5**). Indonesia (n=6,337), Thailand (n=2,747), and the Philippines (n=2,297) had the highest incidence. The same countries recorded the most deaths: Indonesia (n=5,405), Thailand (n=2,490), and the Philippines (n=1,969). Timor-Leste and Brunei had the lowest incidence (Timor-Leste, n=31; Brunei, n=18) and deaths (Timor-Leste, n=18; Brunei, n=12). Lao and Timor-Leste had 100% mortality. In terms of DALYs, Indonesia (n=214,521), the Philippines (n=82,021), and Thailand (n=75,290) still ranked the highest. Singapore (n=2,392), Lao (n=5,481), and Timor-Leste (n=771) had the lowest DALYs (**Table 5**).

**Table 5:** SEA brain tumor burden of disease and bibliometric indices correlational analysis.

Socioeconomic Factors		Bibliometric Indices	Correlation Coefficient (*R*)	*P*-value
***Incidence***		Total Publications	−0.0497	***0.0424^‡^***
Indonesia	6,337			
Thailand	2,747	Plum X Citations	−0.1594	0.1637
Philippines	2,297			
Vietnam	1,452	Plum X Usage	−0.1799	0.5982
Myanmar	1,121			
Malaysia	598	Plum X Captures	−0.1444	0.8264
Cambodia	263			
Singapore	216	Plum X Mentions	−0.2155	***0.0369^‡^***
Lao PDR	113			
Brunei	31	Plum X Social Media	−0.1656	***0.0468^‡^***
Timor-Leste	18			
Total	15,194	Scopus Citations	−0.1469	0.3280
***Death***		Total Publications	−0.0867	***0.0362^‡^***
Indonesia	5,405			
Thailand	2,490	Plum X Citations	−0.1946	0.1801
Philippines	1,969			
Myanmar	1,580	Plum X Usage	−0.2211	0.5407
Vietnam	1,384			
Malaysia	431	Plum X Captures	−0.1813	0.7438
Cambodia	276			
Lao PDR	113	Plum X Mentions	−0.2512	***0.0305^‡^***
Singapore	74			
Timor-Leste	18	Plum X Social Media	−0.2059	***0.040^‡^***
Brunei	12			
Total	13,752	Scopus Citations	−0.1821	0.3793
***DALYs***		Total Publications	−0.1349	***0.0341^‡^***
Indonesia	214,521			
Philippines	82,021	Plum X Citations	−0.2088	***0.0356^‡^***
Thailand	75,290			
Myanmar	59,451	Plum X Usage	−0.2338	***0.0449^‡^***
Vietnam	49,913			
Malaysia	16,258	Plum X Captures	−0.2054	***0.0414^‡^***
Cambodia	11,411			
Lao PDR	5,481	Plum X Mentions	−0.2493	***0.0340^‡^***
Singapore	2,393			
Timor-Leste	771	Plum X Social Media	−0.2317	***0.0342^‡^***
Brunei	506			
Total	518,016	Scopus Citations	−0.2014	***0.0366^‡^***

DALYs, daily adjusted life years; SEA, Southeast Asia.

^‡^Correlation is significant at the 0.05 level (two-tailed).

### Association Analyses Between Socioeconomic Determinants and Burden of Disease Measures With Bibliometric Indices

Population, GDP per capita, %GDP for R&D, and total neurologists did not show any significant correlation with bibliometric indices at *p* value <0.05, except for a negative correlation with total neurologists and mentions (*p* value=0.024). The number of population per neurologist negatively correlated with all bibliometric indices. However, a significant correlation at *p* value <0.1 was noted between: a) population and mentions (*p* value=0.093); b) %GDP for R&D and total publications (*p* value=0.07); and c) total neurologist and total publications (*p* value=0.064) (**Table 2**).

The burden of disease measures showed a negative correlation with bibliometric indices at *p* value <0.05, specifically death with total publications (*p*=0.0362), mentions (*p*=0.0305), and social media (*p*=0.0408). Incidence also showed negative correlation with total publications (*p*=0.0424), mentions (*p*=0.0369), and social media (*p*=0.0468). PlumX indices [total publication (*p*=0.0341), citations (*p*=0.0356), usage (*p*=0.0449), captures (*p*=0.0414), mentions (*p*=0.0340), social media (*p*=0.0342)] and Scopus citations (*p*=0.0365)] were all negatively correlated with DALYs (**Table 3**).

## Discussion

Published reports from SEA on primary CNS tumors from 1991 to 1995 were scarce and were mostly case reports on clinical experience (**Figure 2**), which may support epidemiologic data that CNS tumors are rare in comparison to other neoplasms especially in Asia (15, 22). From 2000 onwards, majority of the published articles focused on the diagnosis and treatment outcomes of GBMs. Though prognosis of brain cancer like GBM is dependent on histology and molecular biology, survival rates vary across continents even for the same tumor type and grade (6). Thus, there is a growing need for evidence-based medicine that takes into consideration geographical, ethnic, and sociocultural differences (3).

The top academic institutions from Singapore, Thailand, and Malaysia dominated the objective measurements of scientific research impact. This trend has been consistently reported for other neurologic diseases like epilepsy, dementia, and multiple sclerosis and neuromyelitis optica (21, 23, 24). The predisposition of these countries to perform well in terms of scientific research and development may be attributed to two reasons. First is their knowledge-based economies that give premium to technology and skill development, thus prioritizing scientific research output, in contrast to agricultural-based economies (23). Second is how developing nations generally lack healthcare systems, which have higher government subsidization and substantial public-private partnerships. This lessens the financial burden on patients, thus increasing access to otherwise costly diagnosis and treatment. When more patients are accurately diagnosed with CNS tumors and subsequently be started on treatment, then more data will be available for research purposes particularly on clinical outcomes (25, 26).

The published evidence regarding correlation of socioeconomic factors with research output about neurologic diseases show that the more developed countries measured by high GDP per capita and those countries that allocate a bigger percent of their GDP to R&D generally produce more research output that do well in terms of traditional and alternative bibliometrics (21, 23, 24). Our study presents a different trend in terms of brain tumor research from SEA countries. GDP per capita did not show any statistically significant correlation with bibliometric indices.

Spending on R&D may not be an appropriate metric in predicting improvement in research output for less common diseases like CNS tumors. We cite three explanations to support this hypothesis. First, primary CNS neoplasms are uncommon in a global scale as well as in the Asian population (3, 22). The fact that even high-income economies like Singapore, wherein molecular tests for diagnosis are available did not show an association of increasing R&D for CNS tumors may reflect the inherent difficulty to generate publications on brain tumors due to its rarity as a disease entity. Second, the diagnosis of primary brain tumors rely heavily on immunohistochemical and molecular tests, which are not readily accessible especially in low-income and developing countries. Theoretically, countries with higher GDP per capita dedicated to R&D should produce more research, however our results could be indicative that looking into the contribution of Total Health Expenditure to GDP could be a better measure for determining association with research output since diagnosis and treatment of brain cancer requires costly advanced methods. Lastly, life expectancy of malignant brain tumors, though improving, is still dismal allowing less time for adequate patient recruitment and selection for research. This discrepancy in terms of epidemiology and burden of disease becomes a hurdle for appropriate attention and funding for neurooncological diseases, which consequently could manifest as inadequacy in research performance from SEA countries.

In terms of socioeconomic determinants, only direct allocation in R&D had the biggest impact in increasing research productivity for primary brain tumors. This direct association of increased R&D spending with improved research output seen in both low and high-income SEA countries was also seen in multiple sclerosis, dementia, and movement disorders (21, 24, 27). This underscores the necessity for investment in research ventures dedicated to CNS neoplasms. The population of SEA countries did not show significant correlation with research output impact metrics. This was in concordance with correlational analyses of SEA studies for multiple sclerosis and dementia (21, 24). In addition, the number of neurologist seem to be negatively correlated to total publications and the number of times the studies get mentioned online. More neurologists did not seem to boost scientific research productivity in the field of CNS tumors.

Aside from increased direct spending on R&D, developing and strengthening human resources and access to care in neuro-oncology may translate to improved research outcomes. Possible strategies include increasing neuro-oncology fellowship opportunities in SEA, access to training programs in western countries, and soliciting additional government support through policymaking. The establishment of the Thai Brain Tumor Society in Thailand, enactment of National Integrated Cancer Control Act in the Philippines, and the Indian Society of Neuro-Oncology Annual Awards and Training Fellowships in Basic, Translational, and Clinical Neuro-Oncology could serve as a foundation for development of a regional collaborative brain tumor research network (8, 28, 29).

Interestingly, the burden of disease had a negative correlation research productivity. This mirrored trends in population size and GDP wherein higher populated countries with low GDP generally had lower research productivity. This trend of low GDP translating into low research output could possibly negatively impact future research ventures into topics on Neuro-oncology in SEA, which could potentially further neglect the unmet needs of these patients. Countries with bigger population and poorer economies would tend to have more cases of brain tumor patients with less access to quality healthcare, therefore resulting in higher mortality. These same countries allocate less of their GDP to R&D and subsequently fair worse in bibliometric indices.

The need to do research for the diagnosis, treatment, and quality of life of neurooncological patients increases as technological advances continue to prolong their survival. CNS cancer has increasing incidence and caused significant morbidity and mortality in the last global report for disease burden (1, 30). There have been previously published neurology-based bibliometric studies (31–37). These concentrated on analysis of highly cited articles, which inadvertently had a selection bias for studies published involving subjects and authors from western countries. Thus, published studies particularly focusing on the Asian population is lacking. To our knowledge, this is the first bibliometric analysis to address the knowledge gap in assessing research productivity output in SEA for primary CNS tumors.

Our study has several limitations. We only included peer-reviewed and published articles as these were readily accessible. Unpublished data from studies presented during proceedings in conferences and from the grey literature were not included, which may affect data on total publication. Another limitation was how search terms used may be too general that may miss specific terms pertaining to each tumor type. One important limitation of this study was to account for the foreseeable economic backlash (i.e. decrease in GDP) that worldwide measures for safety (i.e. quarantines, travel restrictions) will bring about due to the COVID-19 pandemic. The results of this study does not take into consideration the possible acute drop in GDP for SEA countries due to the COVID-19 pandemic. Further, the COVID-19 pandemic may also increase deaths as a metric, as cancer patients are immunocompromised and are a vulnerable population (38). Nevertheless, we employed an exhaustive and systematic search of literature from medical electronic databases.

Based on data presented, countries in the SEA have an increasing incidence of primary brain tumors that are causing significant burden of disease. However, financial and manpower resources to further advance research and development in this area of neurooncology seem to be inadequate based on limited scientific research productivity indices. More attention should be directed in this endeavor especially in recent events of how the COVID-19 pandemic has affected treatment of patients with brain tumors (39). The research performance of SEA countries can be improved by the following: a) increasing allocation of % GDP to research and development; b) strengthening the healthcare system with policies that push for greater government subsidy and increased public and private sector partnerships (26); and c) establishment and promotion of neurological training in residency and fellowship towards a career in neuro-oncology.

## Conclusion

Research output from SEA on primary CNS tumors have been steadily increasing particularly regarding gliomas. Most articles are case reports on clinical experience. High quantity and quality studies came mostly from Singapore, Thailand, and Malaysia. Our study reaffirmed the direct positive correlation of greater percent allocation of GDP to research and development with better research productivity. The burden of disease and total neurologists inversely correlated with bibliometric indices of brain tumor publications. This highlights the importance of increasing public and private resources into producing high-grade publications in neuro-oncology to fill in the gap in the care of patients suffering from primary CNS neoplasms in SEA.

## Data Availability Statement

The original contributions presented in the study are included in the article/supplementary materials; further inquiries can be directed to the corresponding author.

## Author Contributions

MM, AE, and RJ onceptualized the study, contributed to the data curation, conducted a formal analysis, interpreted the data, wrote the original draft, and wrote, reviewed, and edited the manuscript. All authors contributed to the article and approved the submitted version.

## Conflict of Interest

The authors declare that the research was conducted in the absence of any commercial or financial relationships that could be construed as a potential conflict of interest.
